# *catena*-Poly[[bis­(1*H*-indole-5-carboxyl­ato-κ^2^ *O*,*O*′)zinc(II)]-μ-4,4′-azobi­pyridine-κ^2^ *N* ^1^:*N* ^1′^]

**DOI:** 10.1107/S2414314621005228

**Published:** 2021-05-21

**Authors:** Udhayasuriyan Sathya, Jeyaraman Selvaraj Nirmal Ram, Sundaramoorthy Gomathi, Samson Jegan Jennifer, Ibrahim Abdul Razak

**Affiliations:** a Centre for Research and Development, PRIST Deemed to be University, Thanjavur, 613 403, Tamil Nadu, India; b Department of Chemistry, Periyar Maniammai Institute of Science and Technology, Thanjavur 613 403, Tamil Nadu, India; c X-ray Crystallography Unit, School of Physics, University Sains Malaysia, 11800, USM, Penang, Malaysia; Goethe-Universität Frankfurt, Germany

**Keywords:** crystal engineering, coordination polymer, metal-organic framework, structural chemistry, non-covalent inter­actions, crystal structure

## Abstract

The asymmetric unit of the title coordination polymer [Zn(C_9_H_6_NO_2_)_2_(C_10_H_8_N_4_)]_*n*_, consists of one Zn^II^ cation, one bidentate 1*H*-indole-5-carboxyl­ate (I5C) anion and half of a 4,4′-azobi­pyridine (Abpy) neutral ligand. In the coordination polyhedron, the Zn^II^ ion adopts a distorted octa­hedral geometry. The coordination polymer is stabilized by a combination of N—H⋯O and C—H⋯π inter­actions, which leads to the formation of wave-like two-dimensional coordination polymeric layers.

## Abstract 2

The asymmetric unit of the title coordination polymer [Zn(C_9_H_6_NO_2_)_2_(C_10_H_8_N_4_)]_*n*_, consists of one Zn^II^ cation, one bidentate 1*H*-indole-5-carboxyl­ate (I5C) anion and half of a 4,4′-azobi­pyridine (Abpy) neutral ligand. The coordination polymer is stabilized by a combination of N—H⋯O and C—H⋯π inter­actions, which leads to the formation of wave-like two-dimensional coordination polymeric layers.

## Graphical abstract

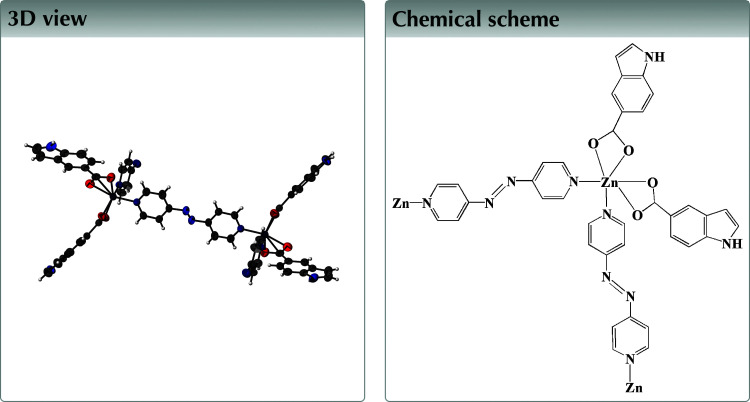

## Structure description

The design of coordination polymers (CPs) and metal–organic frameworks (MOFs) is one of the most important fields in inorganic crystal engineering and material science because of their utility, functions and inter­esting architectures (Ying *et al.*, 2015 ▸; Li *et al.*, 2018 ▸). The self-assembly of metal–organic frameworks and coordination polymers is obtained by complexing metal ions with organic ligands (Li *et al.*, 2018 ▸). In the field of storage and separation sciences, MOFs are a strong competitor for zeolites and carbon nanotubes (Naik *et al.*, 2011 ▸; Cui *et al.*, 2014 ▸). Several MOF structures with Zn^II^ ions have recently been reported (Ying *et al.*, 2015 ▸; Huang *et al.*, 2015 ▸; Liu *et al.*, 2017 ▸; Chen *et al.*, 2020 ▸). In the present work, we report the crystal structure of a Zn^II^-containing coordin­ation polymer constructed using 4,4′-azo­pyridine and indole-5-carb­oxy­lic acid.

**Figure 1:**
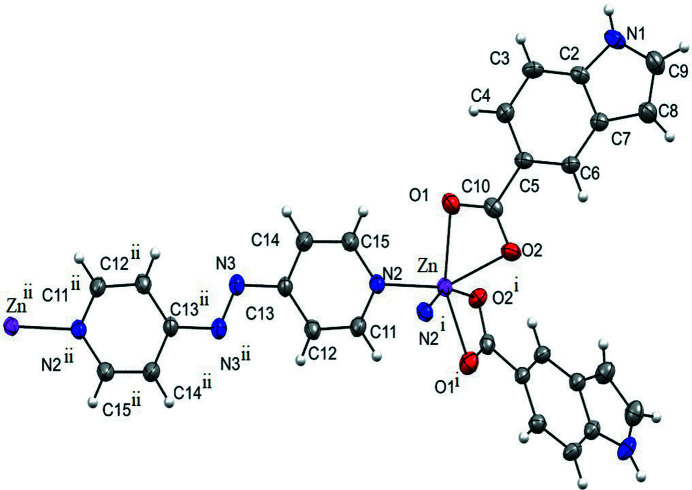
The title complex showing the local coordination around the Zn^II^ metal. Displacement ellipsoids are drawn at the 50% probability level. Symmetry codes: (i) −*x*, *y*, 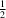 − *z*; (ii) 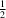 − *x*, 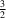 − *y*, 1 − *z*.

The asymmetric unit consists of one Zn^II^ cation, one bidentate 1*H*-indole-5-carboxyl­ate (I5C) anion and half of a 4,4′-azobi­pyridine (Abpy) neutral ligand. The other half of the Abpy ligand is generated by a centre of inversion (symmetry operation − 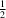 − *x*, 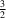 − *y*, −*z*) and it bridges the adjacent Zn^II^ ion as shown in Fig. 1 ▸. Thus, one neutral Abpy ligand bridges two Zn^II^ ions. Each of the Zn^II^ centres has a six-coordinate N_2_O_4_ environment being bonded to the O atoms of two bidentate (I5C) anions and the N atoms of two (Abpy) ligands in a distorted octa­hedral geometry. The Zn—O1, Zn—O2 and Zn—N2 distances are 2.145 (3), 2.227 (3) and 2.098 (3) Å, respectively.

**Figure 2:**
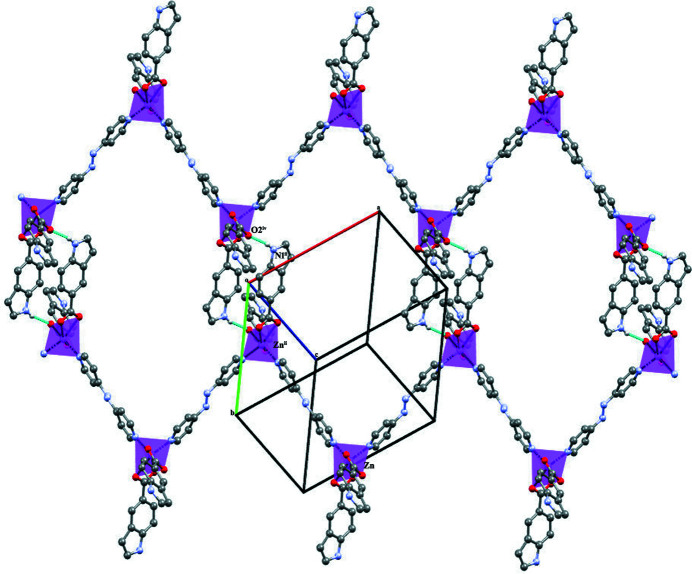
Polyhedral representation of the one-dimensional coordination polymer linked together by N—H⋯O (blue dotted lines).

The six-coordinated monomeric Zn^II^ unit extends as a zigzag chain in the [ 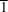 01] direction. Adjacent chains are linked through N—H⋯O^i^ [symmetry code: (i) *x*, −*y*, − 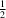 + *z*] hydrogen bonds connecting the N atom of an indole moiety and an O atom of a symmetry-related indole moiety (Table 1 ▸, Fig. 2 ▸). Adjacent layers are held together by weak C—H⋯π inter­actions between the C—H group of an Abpy ligand and the aromatic ring of an I5C anion (Table 1 ▸).

## Synthesis and crystallization

**Figure 3:**
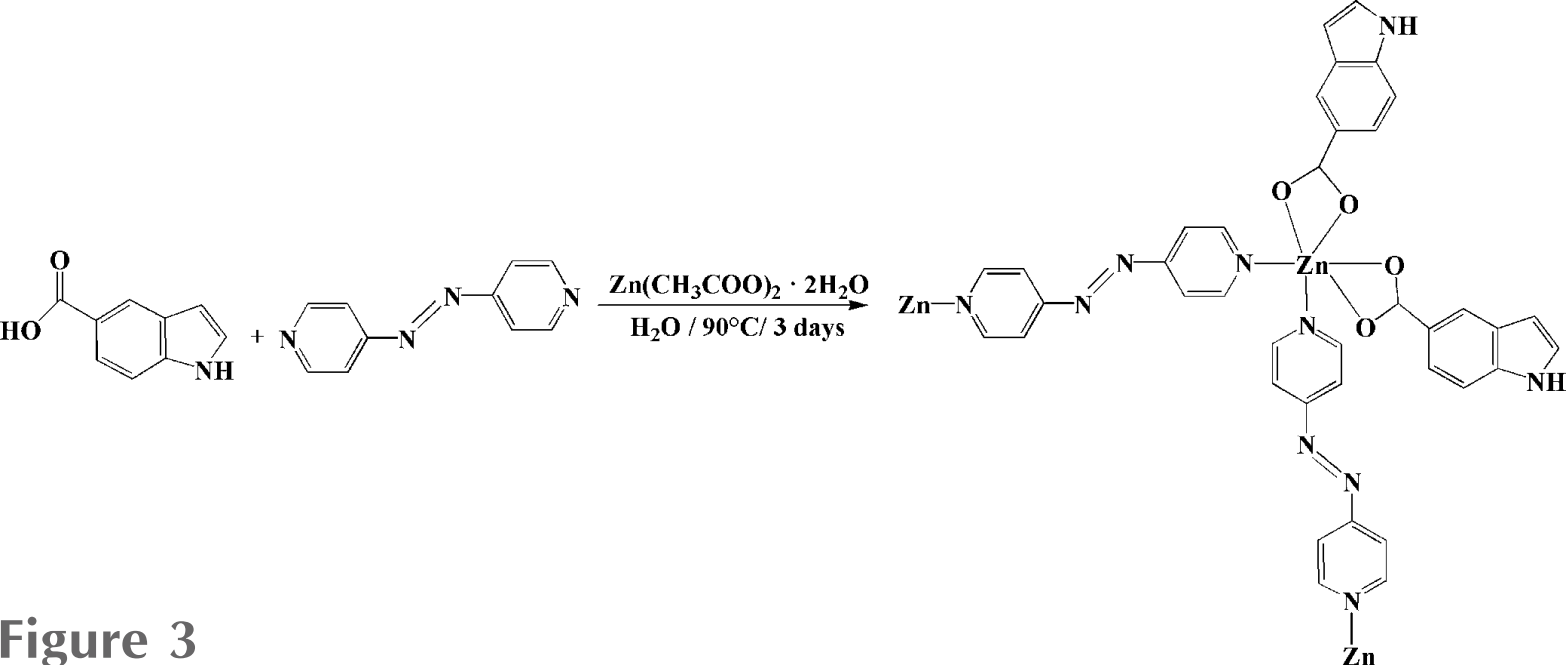
Reaction scheme.

Zn(CH_3_COO)_2_(H_2_O)_2_ (50 mg), indole-5-carb­oxy­lic acid (35 mg), 4,4′-azo­pyridine (35 mg) and deionized water (2.5 ml) were loaded into a 25 ml Teflon-lined stainless steel autoclave to produce the title complex. After being heated at 90°C for 3 d, the autoclave was then cooled to room temperature. Orange–yellow needle-shaped crystals suitable for X-ray diffraction studies were obtained in 65% yield based on the initial Zn(CH_3_COO)_2_(H_2_O)_2_ input. The reaction scheme is shown in Fig. 3 ▸

## Refinement

Crystal data, data collection and structure refinement details are summarized in Table 2 ▸.

## Supplementary Material

Crystal structure: contains datablock(s) I. DOI: 10.1107/S2414314621005228/bt4113sup1.cif

Structure factors: contains datablock(s) I. DOI: 10.1107/S2414314621005228/bt4113Isup2.hkl

CCDC reference: 2083957

Additional supporting information:  crystallographic information; 3D view; checkCIF report

## Figures and Tables

**Table 1:** Hydrogen-bond geometry (Å, °) *Cg* is the centroid of the C2–C7 ring.

*D*—H⋯*A*	*D*—H	H⋯*A*	*D*⋯*A*	*D*—H⋯*A*
N1—H1⋯O2^i^	0.84 (4)	2.02 (4)	2.840 (5)	165 (4)
C12—H12⋯*Cg* ^ii^	0.93	2.79	3.454 (5)	129

Symmetry codes: (i) 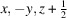 ; (ii) 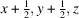 .

**Table 2:** Experimental details

Crystal data	
Chemical formula	[Zn(C_9_H_6_NO_2_)_2_(C_10_H_8_N_4_)]
*M*_r_	569.89
Crystal system, space group	Monoclinic, *C*2/*c*
Temperature (K)	293
*a*, *b*, *c* (Å)	18.982 (3), 11.603 (3), 14.237 (3)
β (°)	119.545 (9)
*V* (Å^3^)	2727.9 (10)
*Z*	4
Radiation type	Mo *K*α
μ (mm^−1^)	0.95
Crystal size (mm)	0.45 × 0.40 × 0.30

Data collection	
Diffractometer	Bruker APEXII CCD
Absorption correction	Multi-scan (*SADABS*; Bruker, 2016 ▸)
*T*_min_, *T*_max_	0.892, 1.000
No. of measured, independent and observed [*I* > 2σ(*I*)] reflections	36639, 3148, 2047
*R*_int_	0.093
(sin θ/λ)_max_ (Å^−1^)	0.652

Refinement	
*R*[*F* ^2^ > 2σ(*F* ^2^)], *wR*(*F* ^2^), *S*	0.057, 0.147, 1.07
No. of reflections	3148
No. of parameters	181
H-atom treatment	H atoms treated by a mixture of independent and constrained refinement
Δρ_max_, Δρ_min_ (e Å^−3^)	0.80, −0.31

Computer programs: *APEX2* and *SAINT* (Bruker, 2016 ▸), *SHELXS97* (Sheldrick 2008 ▸), *SHELXL2018/3* (Sheldrick, 2015 ▸), *PLATON* (Spek, 2020 ▸), *Mercury* (Macrae *et al.*, 2020 ▸), *POVRay* (Cason, 2004 ▸) and *publCIF* (Westrip, 2010 ▸).

## full crystallographic data

### Crystal data

**Table:**

[Zn(C_9_H_6_NO_2_)_2_(C_10_H_8_N_4_)]	*F*(000) = 1168
*M**_r_* = 569.89	*D*_x_ = 1.388 Mg m^−^^3^
Monoclinic, *C*2/*c*	Mo *K*α radiation, λ = 0.71073 Å
*a* = 18.982 (3) Å	Cell parameters from 3148 reflections
*b* = 11.603 (3) Å	θ = 3.0–27.6°
*c* = 14.237 (3) Å	µ = 0.95 mm^−^^1^
β = 119.545 (9)°	*T* = 293 K
*V* = 2727.9 (10) Å^3^	Needle, orange yellow
*Z* = 4	0.45 × 0.40 × 0.30 mm

### Data collection

**Table:**

Bruker APEXII CCD diffractometer	2047 reflections with *I* > 2σ(*I*)
φ and ω scans	*R*_int_ = 0.093
Absorption correction: multi-scan (SADABS; Bruker, 2016)	θ_max_ = 27.6°, θ_min_ = 3.0°
*T*_min_ = 0.892, *T*_max_ = 1.000	*h* = −24→24
36639 measured reflections	*k* = −14→15
3148 independent reflections	*l* = −18→18

### Refinement

**Table:**

Refinement on *F*^2^	0 restraints
Least-squares matrix: full	Hydrogen site location: mixed
*R*[*F*^2^ > 2σ(*F*^2^)] = 0.057	H atoms treated by a mixture of independent and constrained refinement
*wR*(*F*^2^) = 0.147	*w* = 1/[σ^2^(*F*_o_^2^) + (0.0694*P*)^2^ + 3.0748*P*] where *P* = (*F*_o_^2^ + 2*F*_c_^2^)/3
*S* = 1.07	(Δ/σ)_max_ < 0.001
3148 reflections	Δρ_max_ = 0.80 e Å^−^^3^
181 parameters	Δρ_min_ = −0.31 e Å^−^^3^

### Special details

**Table:**

Geometry. All esds (except the esd in the dihedral angle between two l.s. planes) are estimated using the full covariance matrix. The cell esds are taken into account individually in the estimation of esds in distances, angles and torsion angles; correlations between esds in cell parameters are only used when they are defined by crystal symmetry. An approximate (isotropic) treatment of cell esds is used for estimating esds involving l.s. planes.
Refinement. H atoms bonded to C were positioned geometrically and refined using a riding model with C—H = 0.93 and with *U*_iso_(H) = 1.2 *U*_eq_(C). The H atom bonded to N was freely refined.

### Fractional atomic coordinates and isotropic or equivalent isotropic displacement parameters (Å^2^)

**Table:**

	*x*	*y*	*z*	*U*_iso_*/*U*_eq_	
Zn	0.000000	0.32303 (5)	0.250000	0.0404 (2)	
O1	−0.03670 (16)	0.2799 (2)	0.3661 (2)	0.0602 (7)	
O2	−0.08988 (17)	0.1830 (2)	0.2149 (2)	0.0575 (7)	
N2	0.08542 (17)	0.4456 (2)	0.3501 (2)	0.0434 (7)	
N1	−0.1652 (2)	−0.1717 (3)	0.4864 (3)	0.0613 (10)	
C11	0.1396 (2)	0.4866 (3)	0.3247 (3)	0.0526 (10)	
H11	0.142954	0.452255	0.268083	0.063*	
C15	0.0838 (2)	0.4929 (3)	0.4348 (3)	0.0583 (10)	
H15	0.047793	0.463582	0.455364	0.070*	
C10	−0.0757 (2)	0.1944 (3)	0.3109 (3)	0.0469 (9)	
C5	−0.1028 (2)	0.1024 (3)	0.3604 (3)	0.0425 (8)	
C4	−0.0774 (2)	0.1069 (3)	0.4716 (3)	0.0482 (9)	
H4	−0.048102	0.170393	0.511737	0.058*	
C3	−0.0950 (2)	0.0195 (3)	0.5220 (3)	0.0532 (10)	
H3	−0.078181	0.022609	0.595334	0.064*	
C2	−0.1391 (2)	−0.0739 (3)	0.4587 (3)	0.0469 (9)	
C7	−0.1657 (2)	−0.0803 (3)	0.3476 (3)	0.0452 (9)	
C6	−0.1464 (2)	0.0091 (3)	0.2991 (3)	0.0452 (8)	
H6	−0.162844	0.006069	0.225859	0.054*	
C8	−0.2084 (3)	−0.1871 (3)	0.3108 (3)	0.0610 (11)	
H8	−0.232998	−0.215508	0.240609	0.073*	
C9	−0.2062 (3)	−0.2386 (4)	0.3965 (4)	0.0673 (12)	
H9	−0.229238	−0.309784	0.394937	0.081*	
N3	0.23604 (19)	0.7221 (3)	0.5240 (2)	0.0514 (8)	
C13	0.1860 (2)	0.6272 (3)	0.4617 (3)	0.0452 (8)	
C12	0.1904 (2)	0.5767 (3)	0.3778 (3)	0.0513 (9)	
H12	0.227142	0.602936	0.357322	0.062*	
C14	0.1330 (3)	0.5827 (4)	0.4924 (3)	0.0622 (11)	
H14	0.130747	0.613281	0.551185	0.075*	
H1	−0.151 (3)	−0.180 (3)	0.552 (4)	0.063 (13)*	

### Atomic displacement parameters (Å^2^)

**Table:**

	*U*^11^	*U*^22^	*U*^33^	*U*^12^	*U*^13^	*U*^23^
Zn	0.0440 (4)	0.0333 (3)	0.0460 (4)	0.000	0.0237 (3)	0.000
O1	0.0641 (18)	0.0530 (16)	0.0655 (18)	−0.0108 (14)	0.0335 (15)	0.0033 (14)
O2	0.0726 (18)	0.0563 (17)	0.0555 (17)	0.0030 (14)	0.0406 (14)	0.0111 (13)
N2	0.0444 (17)	0.0367 (16)	0.0532 (18)	−0.0036 (13)	0.0272 (15)	−0.0041 (13)
N1	0.064 (2)	0.068 (2)	0.056 (2)	−0.0037 (18)	0.0317 (19)	0.020 (2)
C11	0.059 (2)	0.047 (2)	0.059 (2)	−0.0088 (19)	0.035 (2)	−0.0158 (19)
C15	0.068 (3)	0.056 (2)	0.066 (3)	−0.019 (2)	0.045 (2)	−0.015 (2)
C10	0.0398 (19)	0.045 (2)	0.059 (2)	0.0081 (17)	0.0265 (18)	0.0116 (19)
C5	0.046 (2)	0.046 (2)	0.0423 (19)	0.0089 (16)	0.0267 (17)	0.0100 (16)
C4	0.050 (2)	0.049 (2)	0.046 (2)	−0.0010 (17)	0.0241 (18)	−0.0024 (17)
C3	0.055 (2)	0.069 (3)	0.039 (2)	0.005 (2)	0.0255 (18)	0.0095 (19)
C2	0.044 (2)	0.051 (2)	0.047 (2)	0.0038 (17)	0.0238 (17)	0.0115 (18)
C7	0.046 (2)	0.047 (2)	0.045 (2)	0.0044 (17)	0.0249 (17)	0.0049 (17)
C6	0.050 (2)	0.051 (2)	0.0383 (19)	0.0025 (17)	0.0242 (17)	0.0044 (17)
C8	0.066 (3)	0.058 (3)	0.055 (2)	−0.007 (2)	0.027 (2)	0.005 (2)
C9	0.067 (3)	0.058 (3)	0.072 (3)	−0.008 (2)	0.031 (2)	0.008 (2)
N3	0.0558 (19)	0.0453 (18)	0.0508 (19)	−0.0127 (15)	0.0245 (15)	−0.0079 (15)
C13	0.048 (2)	0.0369 (19)	0.048 (2)	−0.0060 (16)	0.0219 (18)	−0.0058 (16)
C12	0.052 (2)	0.045 (2)	0.066 (3)	−0.0098 (17)	0.036 (2)	−0.0088 (19)
C14	0.081 (3)	0.061 (3)	0.059 (2)	−0.023 (2)	0.045 (2)	−0.020 (2)

### Geometric parameters (Å, º)

**Table:**

Zn—N2	2.098 (3)	C5—C6	1.381 (5)
Zn—N2^i^	2.098 (3)	C5—C4	1.410 (5)
Zn—O1	2.145 (3)	C4—C3	1.375 (5)
Zn—O1^i^	2.145 (3)	C4—H4	0.9300
Zn—O2^i^	2.227 (3)	C3—C2	1.394 (5)
Zn—O2	2.227 (3)	C3—H3	0.9300
Zn—C10^i^	2.505 (4)	C2—C7	1.405 (5)
Zn—C10	2.505 (4)	C7—C6	1.392 (5)
O1—C10	1.255 (4)	C7—C8	1.431 (5)
O2—C10	1.263 (5)	C6—H6	0.9300
N2—C11	1.333 (4)	C8—C9	1.340 (6)
N2—C15	1.340 (4)	C8—H8	0.9300
N1—C9	1.365 (6)	C9—H9	0.9300
N1—C2	1.373 (5)	N3—N3^ii^	1.236 (6)
N1—H1	0.84 (4)	N3—C13	1.438 (5)
C11—C12	1.370 (5)	C13—C12	1.369 (5)
C11—H11	0.9300	C13—C14	1.379 (5)
C15—C14	1.370 (5)	C12—H12	0.9300
C15—H15	0.9300	C14—H14	0.9300
C10—C5	1.502 (5)		
			
N2—Zn—N2^i^	94.68 (15)	C14—C15—H15	118.7
N2—Zn—O1	94.06 (11)	O1—C10—O2	120.3 (3)
N2^i^—Zn—O1	104.19 (11)	O1—C10—C5	120.1 (3)
N2—Zn—O1^i^	104.19 (11)	O2—C10—C5	119.6 (3)
N2^i^—Zn—O1^i^	94.06 (11)	O1—C10—Zn	58.88 (19)
O1—Zn—O1^i^	153.04 (16)	O2—C10—Zn	62.61 (19)
N2—Zn—O2^i^	95.28 (10)	C5—C10—Zn	166.6 (2)
N2^i^—Zn—O2^i^	153.72 (10)	C6—C5—C4	120.4 (3)
O1—Zn—O2^i^	99.28 (10)	C6—C5—C10	119.8 (3)
O1^i^—Zn—O2^i^	59.90 (10)	C4—C5—C10	119.6 (3)
N2—Zn—O2	153.72 (10)	C3—C4—C5	121.4 (3)
N2^i^—Zn—O2	95.28 (10)	C3—C4—H4	119.3
O1—Zn—O2	59.91 (10)	C5—C4—H4	119.3
O1^i^—Zn—O2	99.28 (10)	C4—C3—C2	117.3 (3)
O2^i^—Zn—O2	86.31 (14)	C4—C3—H3	121.3
N2—Zn—C10^i^	104.80 (11)	C2—C3—H3	121.3
N2^i^—Zn—C10^i^	123.50 (12)	N1—C2—C3	130.1 (4)
O1—Zn—C10^i^	125.99 (13)	N1—C2—C7	107.4 (3)
O1^i^—Zn—C10^i^	30.07 (11)	C3—C2—C7	122.5 (3)
O2^i^—Zn—C10^i^	30.23 (11)	C6—C7—C2	118.8 (3)
O2—Zn—C10^i^	89.71 (10)	C6—C7—C8	134.7 (3)
N2—Zn—C10	123.50 (12)	C2—C7—C8	106.5 (3)
N2^i^—Zn—C10	104.80 (11)	C5—C6—C7	119.5 (3)
O1—Zn—C10	30.07 (11)	C5—C6—H6	120.2
O1^i^—Zn—C10	125.99 (13)	C7—C6—H6	120.2
O2^i^—Zn—C10	89.71 (10)	C9—C8—C7	107.1 (4)
O2—Zn—C10	30.23 (11)	C9—C8—H8	126.4
C10^i^—Zn—C10	106.87 (17)	C7—C8—H8	126.4
C10—O1—Zn	91.0 (2)	C8—C9—N1	110.3 (4)
C10—O2—Zn	87.2 (2)	C8—C9—H9	124.8
C11—N2—C15	117.4 (3)	N1—C9—H9	124.8
C11—N2—Zn	119.9 (2)	N3^ii^—N3—C13	113.1 (4)
C15—N2—Zn	122.4 (2)	C12—C13—C14	118.9 (3)
C9—N1—C2	108.7 (4)	C12—C13—N3	124.1 (3)
C9—N1—H1	134 (3)	C14—C13—N3	117.0 (3)
C2—N1—H1	117 (3)	C13—C12—C11	118.6 (3)
N2—C11—C12	123.4 (3)	C13—C12—H12	120.7
N2—C11—H11	118.3	C11—C12—H12	120.7
C12—C11—H11	118.3	C15—C14—C13	119.0 (4)
N2—C15—C14	122.6 (4)	C15—C14—H14	120.5
N2—C15—H15	118.7	C13—C14—H14	120.5

Symmetry codes: (i) −*x*, *y*, −*z*+1/2; (ii) −*x*+1/2, −*y*+3/2, −*z*+1.

### Hydrogen-bond geometry (Å, º)

*Cg* is the centroid of the C2–C7 ring.

**Table:**

*D*—H···*A*	*D*—H	H···*A*	*D*···*A*	*D*—H···*A*
N1—H1···O2^iii^	0.84 (4)	2.02 (4)	2.840 (5)	165 (4)
C12—H12···*Cg*^iv^	0.93	2.79	3.454 (5)	129

Symmetry codes: (iii) *x*, −*y*, *z*+1/2; (iv) *x*+1/2, *y*+1/2, *z*.
